# Endometrial Stem Cells in Farm Animals: Potential Role in Uterine Physiology and Pathology

**DOI:** 10.3390/bioengineering5030075

**Published:** 2018-09-18

**Authors:** Evelyn Lara, Nathaly Rivera, Joel Cabezas, Felipe Navarrete, Fernando Saravia, Lleretny Rodríguez-Alvarez, Fidel Ovidio Castro

**Affiliations:** Department of Animal Science, Faculty of Veterinary Sciences, Universidad de Concepción, Chillán 3780000, Chile; evlara@udec.cl (E.L.); nathrivera@udec.cl (N.R.); jocabezas@udec.cl (J.C.); fnavarreteaguirre@gmail.com (F.N.); fsaravia@udec.cl (F.S.); llrodriguez@udec.cl (L.R.‐A.)

**Keywords:** mesenchymal stem cells, endometrium, livestock

## Abstract

The endometrium is an accessible source of mesenchymal stem cells. Most investigations of endometrial mesenchymal stem cells (eMSCs) have been conducted in humans. In animals, particularly in livestock, eMSC research is scarce. Such cells have been described in the bovine, ovine, caprine, porcine, and equine endometrium. Here we provide the state of the art of eMSCs in farm animals with a focus on the bovine species. In bovines, eMSCs have been identified during the phases of the estrous cycle, during which their functionality and the presence of eMSC-specific markers has been shown to change. Moreover, postpartum inflammation related to endometritis affects the presence and functionality of eMSCs, and prostaglandin E_2_ (PGE_2_) may be the mediator of such changes. We demonstrated that exposure to PGE_2_ in vitro modifies the transcriptomic profile of eMSCs, showing its potential role in the fate of stem cell activation, migration, and homing during pathological uterine inflammation in endometritis and in healthy puerperal endometrium. Farm animal research on eMSCs can be of great value in translational research for certain uterine pathologies and for immunomodulation of local responses to pathogens, hormones, and other substances. Further research is necessary in areas such as in vivo location of the niches and their immunomodulatory and anti-infective properties.

## 1. Introduction

Stem cells are undifferentiated cells defined by their potency and ability to differentiate into other specific cell types [1]. After birth, these cells are scarce, nevertheless they can be found in bone marrow, umbilical cord, and associated tissues, fat, and blood among other adult tissues, where they display limited proliferation potential and are named adult, somatic or tissue-specific stem cells [2]. Stem cells play a key role in tissue homeostasis and integrity, due to their ability to maintain, generate and replace terminally differentiated cells as part of physiologic cell renewal or after tissue damage [3]. The endometrium is a highly regenerative tissue that undergoes diverse cell proliferation, growth, and apoptosis cycles as a function of the estrous cycle, pregnancy and involution or uterine pathologies [4,5]. During the estrous cycle in most animals, the endometrium shows periods of cell growth, apoptosis, and endometrial loss, without exhibiting bleeding [6]. Regulation of these changes by sex hormones, which are key factors in tissue regeneration controlled by mitogenic effects and/or stromal and epithelial cell differentiation during both the estrous cycle and pregnancy, has been described [7]. Undoubtedly, the endometrium bears great cell-renewal ability, similar to what is observed in highly regenerative tissues such as hematopoietic bone marrow, epidermis, and intestinal epithelium, in which stem cells are responsible for continuous cell production and regeneration [6]. It is now generally accepted that endometrial remodeling is mediated by specialized cells, such as stem cells, that reside in the uterine compartment [8]. 

In particular, it has been proposed that endometrial stem cells located in or migrating to the uterus are responsible for endometrial regeneration; however, their origin, exact anatomical location and clear biological functions has remained elusive, and endometrial repopulation from an individual clonal endometrial stem cell has not been achieved thus far [9]. The first evidence of the existence of endometrial stem cells was demonstrated in humans [10]. Since then, tens of endometrial stem cell studies have been reported, mainly in human and murine models; however, information available regarding other species, such as farm animals, is scarce. Nevertheless, stem cells have been described in or isolated from the endometrium of diverse species such as porcine [11], bovine [12], ovine [13], caprine [14] and equine [15].

## 2. Functional Morphology of Uterine Compartments

The uterus is a tubular organ with a wall and lumen. The uterine wall is covered by an external layer of serosa facing the peritoneum (perimetrium), while inner tissues are grouped in the myometrium, composed of a thick layer of smooth muscle cells and the glandular mucosal layer that has direct contact with the lumen of the uterus: the endometrium. The latter is composed of a functional and a basal zone, in turn the functional zone is divided into a compact stratum and a spongy layer. The functional zone degenerates totally or partially during menstruation in humans. The basal zone is thin and persists throughout the cycle. When the functional zone is lost, it is regenerated from this layer, where most likely reside stem cell niches [16,17,18]. The endometrium-myometrial interface lacks a layer of intermediate tissue, and therefore the myometrium is vulnerable to invasion by the endometrium [19]. The cellular components of the endometrium include the luminal and glandular epithelium, stroma, endothelium, and some cells of the immune system [20]. The main biological function of the uterine glands is the secretion of the histotrophic fluid, which has an important role in the supply of nutrients during embryo implantation and early fetal development [21]. The stroma is dense and contains large number of blood vessels [19,20,21] and it is composed mainly of fibroblasts that deposit extracellular matrix, and resident and migratory cells such as macrophages, lymphocytes, and eosinophils [19]. 

As opposite to the highly columnar endometrial architecture present in primates, including humans, in farm animals as a rule, the endometrium has a uniform structure; the luminal epithelium is surrounded by highly vascularized stroma in which the endometrial glands are located. Glands vary in their shape and number, according to the species [22]. In the endometrium of ruminants, there is a glandular (caruncular) area where takes place the exchange of gases and micronutrients between the pregnant female and the fetus. The intercaruncular (glandular) regions correspond to the place where the endometrial glands are located [23]. 

In mares, the functional area of the endometrium is lined by a cuboidal secretory epithelium seated in the basement membrane and the lamina propria. In this segment it is possible to locate blood and lymphatic vessels [24]. The stromal cells are oval, with a large nucleus and scarce cytoplasm, in the compact stratum they are in great density, the spongy stratum, on the other hand, has few scattered cells [24]. The myometrium is formed by a thick internal circular layer and a thin external longitudinal layer, among them is the vascular layer [16].

## 3. Role and Biology of Endometrial Stem Cells

Stem cells are rare in adult tissues other than bone marrow and lack distinctive morphologic characteristics, which makes them difficult to locate; therefore, their identification is mainly based on the study of their functional properties [25]. Published studies about the identification of stem cells in the endometrium have revealed a great deal of information regarding possible mechanisms involved in endometrial regeneration in mammals [26]. Consequently, various theories on the origin of endometrial stem cells have been proposed, such as derivation from residual fetal stem cells, which can persist until the postmenopausal period and are preferentially localized in the basal layer of the tissue stroma [27]. On the other hand, various studies have focused on the existence of an extra-uterine source of adult stem cells, which presumably could be recruited towards the endometrial tissue in each cycle, either from perivascular cells [28] or bone marrow [29]. However, it has been suggested that, although bone marrow-derived stem cells can be the origin of endometrial stem cells, it cannot be assured that they are the source of stem cells responsible for endometrial regeneration during each cycle [17]. There is yet another possibility that does not preclude the above-mentioned potential origins of endometrial stem cells: endometrial stem cells resident in the epithelium and stroma are responsible for cyclic cellular regeneration in this tissue [10].

Despite their potential external origin, endometrial stem cells per se also have migratory capability. However, to date, comparative studies assessing the migratory characteristics of bone marrow MSC (mesenchymal stem cell) with their assumable endometrial progeny, had not been performed for any farm animal species. In humans, such comparison showed that upon cell migration and change in niche, the cytokine secretion profile and thereby paracrine signaling is also changed [30], thus MSC acquire a niche-dependent pro- and anti-inflammatory properties, which also alters their migration [31], specific surface marker [32] and inflammation driven migration profile for the bmMSCs, eMSCs and endometrial stromal fibroblasts [17].

We showed that bovine and equine endometrial MSC migrate towards attractants using both scratch and transwell assays [12,15] and that in mares, this migration ability was niche-dependent. The migration pattern was significantly faster for adipose MSC than for endometrial MSC obtained from the same donors. When next generation sequence of mRNAs from both cell types was performed, several genes involved in chemo attraction were down-regulated in endometrial MSC, particularly dramatic was the under expression of CXCL8 (interleukin 8; IL8) and platelet derived growth factor D, both important genes involved in cell migration (manuscript in preparation). 

As quoted in [1]: “stem cells are not only units of biological organization which are responsible for development and regeneration of both organ and tissue, but also units of natural selection evolution”. It was observed that, under certain micro environmental conditions, these cells may change their destiny to cell types other than those of their original tissue source, showing remarkable cell plasticity and differentiation ability [33]. Endometrial stem cells have manifested their great ability to differentiate into adipocytes, osteocytes, chondrocytes, myocytes, and endothelial cells [34]. Moreover, it was reported that endometrial stem cells might differentiate into neural, pancreatic, hepatic, epithelial and cardiac lineages [35]. The plasticity characteristics of endometrial cells are thus similar to what was observed for bone marrow and adipose tissue stem cells, which have been used in diverse studies of tissue reconstruction and have been suggested as an alternative to regenerative therapy in tissue [36].

Mesenchymal stem cells from endometrium (eMSCs) have been the subject of intensive research in endometrium stem cell biology due to their potential application in regenerative therapy, migration and nesting in tissue, promotion of angiogenesis and prevention of fibrosis and apoptosis, as well as for their immunosuppressive and anti-inflammatory properties [37].

The endometrium has been considered as an attractive, accessible, and renewable source of MSCs [6]. These cells can be isolated easily from tissue by endometrial biopsy, which is obtained non-invasively and without the need for anesthesia [38]. All these characteristics make eMSCs an interesting option for use in regenerative therapy, in the reproductive tract as well as in other tissues [39,40]. 

## 4. Stem Cells in the Endometrium of Farm Animals

During each estrous cycle, the endometrium undergoes the processes of morphological and functional modification, giving rise to endometrial remodeling, angiogenesis and invasive growth regulation, cell adhesion and embryo feeding [41]. It is, therefore, tempting to postulate that eMSCs are responsible for tissue regeneration in key periods in the female’s life [42]. In farm animals, different stem cell candidates have been identified in endometrial tissue, but available studies are scarce in some of these species (Table 1).

In 2008, bovine endometrial stromal cells were isolated and cultured for the first time, showing spontaneous morphological change, similar to the bovine stromal bone marrow cell phenotype after 18 days in culture. In addition, these cells differentiated into the osteogenic lineage, and the authors proposed the presence of progenitor-like mesenchymal endometrial cells [45]. Our research group showed, for the first time, the presence of progenitor stem cells in bovine endometrium during early and late phases of the estrous cycle [12]. These cells were identified according to their specific characteristics, such as adherence to plastic, a high proliferation rate and the ability to form colonies and differentiate into chondrogenic and osteogenic lineages, in addition to demonstrating the expression of both multipotent and pluripotent stem cell markers such as STAT3, CD44, c-KIT, OCT4 and SOX2, both at the mRNA and protein levels.

In another study, [46], NANOG was detected among expressed markers in bovine eMSC, likewise expression of C-KIT, OCT3/4, NANOG and SOX2 in both endometrial and myometrial cells of cattle was observed in days 5 to 10 of the estrous cycle [47]. Recently, Lara et al., 2017a [48], extended the original findings of [12], to the follicular phase of the estrous cycle in cattle. Again, and in contradiction with [46], NANOG was not detected but f OCT4, SOX2 and several markers of multipotency were.

In sows, Oct4, Sox2 and Nanog have been expressed in an endometrial stromal cell population, which show the ability to self-renew and have a high proliferation score [43]. There is no evidence of the actual pluripotency of these cells, and they most likely play another as yet undetermined function, which is not known at present. Therefore, caution should be used when these markers are claimed as markers of pluripotency, since it is believed that Oct4, Sox2 and Nanog expression is specific to embryonic pluripotent cells, and its regulation is key to self-renewal and maintaining cells in an undifferentiated state [49].

Due to the absence of tissue- and species-specific surface eMSC markers, researchers have focused on the evaluation of functional stem cell characteristics rather than on their surface proteins. In this way, potential eMSCs have been described in the bovine, ovine, caprine, porcine, and equine endometrium [11,13,14,15,50]. These cells adhere to plastic and differentiate in vitro into chondrocytes, osteocytes, and adipocytes at the minimum, in addition to expressing particular membrane markers, some of which have been previously described in humans [40]. 

In the bovine endometrium, the presence of eMSCs was determined by their clonogenicity and differentiation into adipogenic and osteogenic lineages, along with the expression of markers including CD29, CD44, MHC-II and CD34 [12,48]. A better functional evaluation of bovine endometrial MSCs showed that these cells possess fibroblast-like morphology, plastic adherence, high proliferative capacity, clone formation in vitro and the ability to differentiate into chondrogenic, osteogenic and adipogenic lineages [50]

In other species, e.g., ovine, a specific population of CD271^+^ CD49f^−^ was found to display high clonogenic efficiency upon re-cloning and differentiation to adipogenic, myogenic, chondrogenic and osteogenic lineages [13]. In the case of caprine species, eMSCs also showed high proliferation and in vitro differentiation potential, obtaining osteogenic and chondrogenic lineages [14]. In sows the existence of eMSCs was determined based on their high clonogenic ability, both osteogenic and chondrogenic differentiation and the expression of MSCs markers described for humans, such as CD29, CD44, CD144, CD105 and CD140b, as well as the absence of CD34 and CD45 [11]. Finally, in equines, endometrial MSCs have been isolated and characterized. These cells showed a fibroblast-like morphology, grew on plastic, and differentiated into the mesenchymal tri-lineage (osteo-, chondro- and adipogenic). Moreover, they showed rapid population doubling and migrated towards fetal calf serum in a scratch assay, alongside the expression of CD44, CD90 and MHCI surface markers [15]. These properties were compared with those of adipose MSC; in that study, we concluded that equine-derived endometrial MSC, though they share biological attributes with adipose MSCs of this species, display a different surface marker phenotype and an impaired migration ability.

### 4.1. Endometrial Stem Cells during the Oestrous Cycle in Farm Animals: What Do They Show?

Changes occurring in the endometrium during the estrous cycle are regulated by sex hormones, which are key factors in tissue regeneration via their mitogenic effects and/or stromal and epithelial cell differentiation [7]. Likewise, plasma progesterone and estradiol levels mainly rely on ovarian structures present in the different phases of the estrous cycle [51]. During the follicular phase, circulating estrogen levels increase, regulating the survival, viability and mitogenic effects of endometrial cells [52]. On the other hand, during the luteal phase there is a progressive increase in progesterone secreted by the corpus luteum, which generates a dispersed epithelial cell surface, active uterine gland proliferation and high histotrophic secretion [41]. These hormonal variations during the estrous cycle affect and modify endometrial cell behavior, which might affect the presence or characteristics of stem cells residing in different phases [50]. It is believed that endometrial stem cell activity during the estrous cycle is influenced by ovarian hormones at the local level, since stronger expression of molecular markers from these cells was observed in the ipsilateral horn [46]. In goats, MSCs obtained from anestrous endometrium showed a shorter population doubling time than cells obtained from any cyclic stage; this may be related to the increase and accumulation of growth factors in the endometrial tissue during this particular stage [14]. Furthermore, in the case of pigs, it was found that the stage of the cycle determines the percentage of stem or progenitor cells in the endometrium, which is significantly higher at day 19 than at days 2–4 of the cycle [43]. Similarly, cow endometrium showed MSCs with a longer cell doubling time during the early luteal phase in comparison with the follicular and late luteal phases [47]. It is believed that the doubling time of the endometrial MSC population can be affected by two principal factors, namely cyclic steroid hormone levels and aging [45]. In fact, it is proposed that the presence of endometrial stem cells in the bovine endometrium is higher in younger animals than in older ones [46].

The proliferative ability of eMSCs seems to be affected by the phase of the estrous cycle, while cell clonogenicity is not, suggesting that the ability to form colonies is independent of ovarian hormones present in the tissue [11,50]. A more detailed study of bovine endometrium determined that the functionality and presence of markers changed throughout the estrous cycle, between the follicular and both early and late luteal phases [50]. According to these findings, eMSCs seem to exist in cattle endometrium in the follicular and late luteal phases but not during mid-estrous (early luteal phase), where other type of more committed, probably progenitor cells are present [12,48]. In opposition to our findings, another study showed the presence of MSCs in the bovine endometrium during days 5 to 10 (stage II or middle phase) and days 11 to 17 (stage III or late luteal phase) of the estrous cycle and postulated that the characteristics of these cells tend to be similar and independent of the phase of the estrous cycle they belong to [47]. However, the absence of changes in stem cells may be because, from the middle phase on, there is a mature corpus luteum that lasts until the late luteal phase, and the concentrations of progesterone are high and similar between the two stages [53].

**Figure 1:**
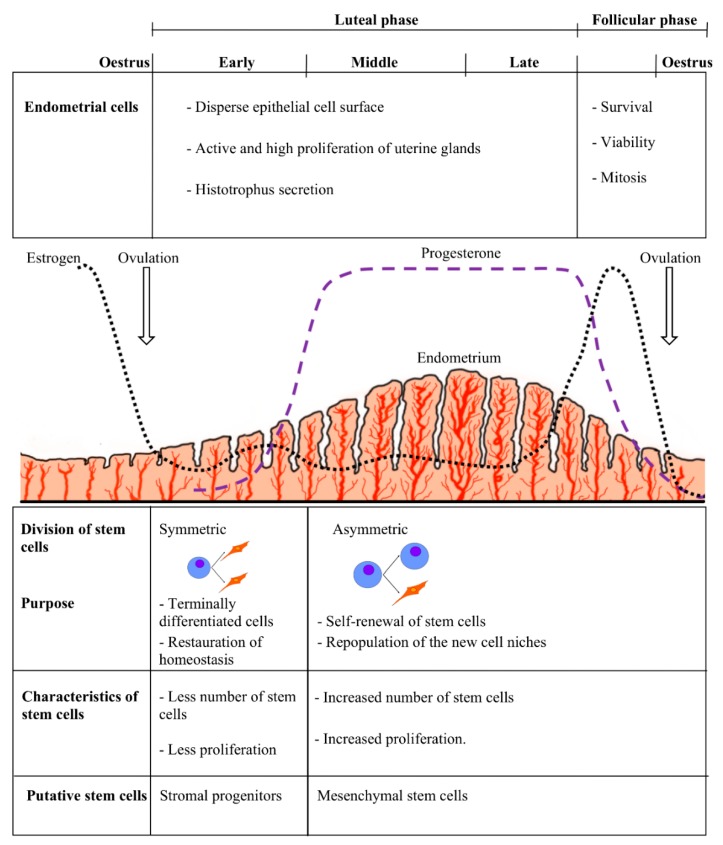
Stem cells and hormonal variation in the estrous cycle, indicating the relation between the type and characteristics of the stem cells with the appropriate stages of the estrous cycle in a hypothetical animal model.

It seems logical that functional characteristics of endometrial stem cells would be similar throughout the estrous cycle but not identical between different stages; this could be explained by differential levels of circulating ovarian hormones as well as by differential responses of dissimilar cell types to those hormones [54]. Furthermore, hormonal regulation can be mediated by paracrine signals resulting from the interaction of progesterone and estrogen with the progenitor cell niche [55]. In humans, endometrial cells shed during the menstrual cycle must be replaced by new cells generated from resident cells while maintaining tissue integrity [56]. Low estrogen and progesterone concentrations seem to stimulate symmetric divisions, which play a key role in the restoration of homeostasis and delivery of terminally differentiated cells [50,57]. Also, these increases in hormones lead to intense stromal cell mitosis, which can stimulate asymmetric division in the cell niche, which in turns leads to self-renewal of stem cells and repopulation of the new cell niches [58] (Figure 1).

### 4.2. Stem Cells and Critical Periods of Endometrial Regeneration in Farm Animals

#### 4.2.1. Endometrial Stem Cells during Pregnancy and Puerperium

During pregnancy and soon after birth, the endometrium undergoes intense cellular changes in both morphology and tissue functionality. Particularly in early postpartum (puerperium), these changes involve tissue repair, apoptosis, proliferation, degradation, and reorganization of the extracellular matrix [59,60]. 

Pregnancy and postpartum are considered as the periods of highest endometrial remodeling, repair, and regeneration [61]. In primates, after partial endometrial resection, complete tissue regeneration has been proven, and females were even able to sustain gestation until 28 weeks of pregnancy [42]. In mice, nesting of hematopoietic progenitor cells in the endometrial luminal epithelium was observed; a colonization rate of 82% of the total of these cells was obtained in the case of pregnancy, and they appeared in the long term at the vascular endothelium level [62]. It is suggested that stem cells may facilitate the endometrium regeneration that takes place immediately after parturition to restore tissue homeostasis [61]. After parturition, uterine involution occurs in a species-specific manner. These changes are intended to recover uterine structure and function for the next pregnancy [63]. 

In the presence of an embryo, progesterone-induced changes impinge upon endometrial morphology and functionality [64]. Literature about the precise cellular endometrial changes that occur during this critical pre-implantation period is scarce due to difficulties associated with sampling and the absence of in vitro models. Conversely, several papers had addressed transcriptomic and/or proteomic changes in the endometrium in the presence/absence of an embryo, particularly in bovines [64,65,66]. Elevated progesterone alters endometrial gene expression in early pregnancy and has been associated with triglyceride synthesis and glucose transport, which may contribute as an energy source for the developing embryo [65]. Likewise, transcriptome studies of endometrial samples recovered during the pre-attachment period identified many interferon-stimulated genes and those possibly involved in embryo-maternal immune modulation, as well as genes affecting cell adhesion and remodeling of the endometrium [64]; more precisely, these changes differ between caruncural and inter caruncural tissue [66].

In the case of puerperal farm animals, the presence of stem cells during these periods has been scarcely studied; only one existing study reported on the presence of stem cells during the bovine postpartum period. In a previous report, we isolated endometrial MSCs from <26 days puerperal healthy cows and showed those cells to have a fibroblast-like morphology, adherence to plastic, high clonogenicity, enhanced proliferative capacity and to differentiate into chondrogenic, osteogenic and adipogenic lineages in vitro [50]. In cows, endometrial repair starts immediately after parturition, and epithelium regeneration in seriously damaged areas such as caruncles is complete in as few as 25 days [67,68]. To our knowledge, there are no other reports about eMSCs from cattle or other animals during this postpartum period.

#### 4.2.2. Stem Cells in the Endometrium in Uterine Pathology

The post-partum endometrium is prone to endometritis caused by pathogenic bacteria acquired during delivery. This pathology alters tissue integrity and can affect the usual endometrial regeneration [69], which may lead to consequences in the reproductive outcome of the female. Tissue damage can evoke MSC migration from other tissues to the endometrium, as well as local regeneration of resident eMSC, which eventually would re-populate the tissue [70,71]. It has been proposed that stromal cells may differentiate into other lineages within the endometrium and are associated with infertility [44].

In bovine adenomyosis there is an increase in the expression of pluripotency markers in both endometrial tissue and in cells derived from dysfunctional tissue compared to control healthy animals [72]. The authors proposed that variation in ovary steroids, particularly estrogen, leads to alteration in the functionality and proliferation of endometrial stem cells, with the consequent development of uterine pathology. Others have also linked estrogen dysregulation in mice with an increase in the specific population of endometrial stem cells after induction of tissue injury [73].

Our research group investigated the potential influence of postpartum inflammation on the presence and functionality of eMSCs. Said cells were isolated from cows diagnosed with subclinical and clinical endometritis as well as from healthy puerperal cows [50]. MSCs isolated from clinically ill cows did not differentiate into adipocytes and showed impaired cloning efficiency and a longer cell doubling time when compared to sub-clinically ill and to healthy postpartum cows. Postpartum uterine infection produces degenerative changes in the endometrium with total or partial tissue loss [69]. Likewise, inflammation can affect progenitor/stem cells directly in the total number of cellular divisions and cause premature senescence, observing a slower growth rate, which might affect cellular differentiation [74]. Tissue damage associated with parturition and inflammation may modulate the physical properties of cell membranes and the shape of cells causing cell death by bacterial pore-forming toxins affecting tissue repair [75]. It has been proposed that, in chronic injury or aging, there exists limited self-renewal of stem cells, resulting in restricted regenerative ability of a tissue [76].

#### 4.2.3. Effects of Different Effector Molecules on Endometrial Stem Cells

**Figure 2:**
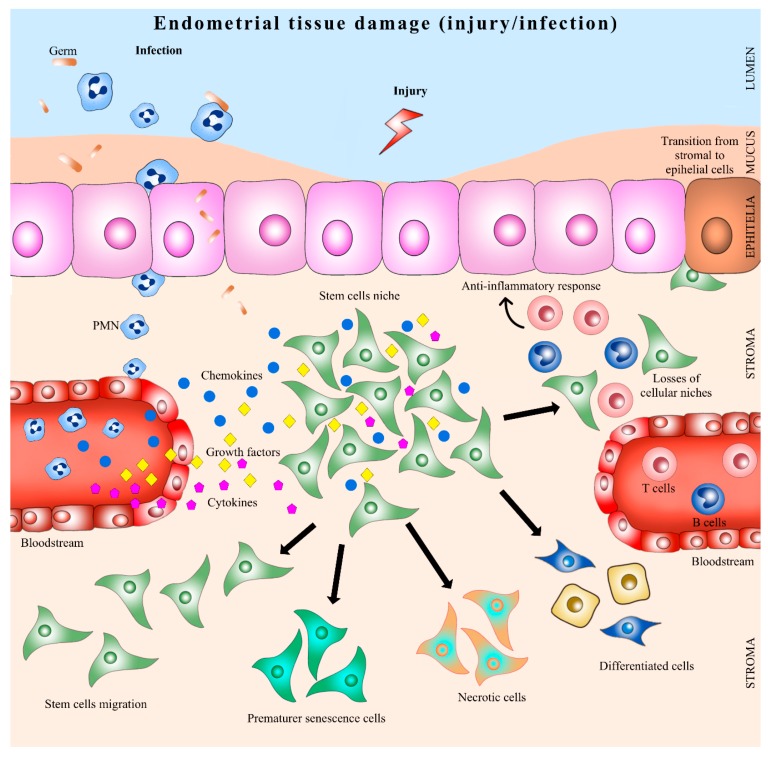
Endometrial stem cells in uterine inflammation. PMN: polymorphonuclear neutrophil.

Infection and inflammation might inhibit regeneration of traumatized endometrium by effector molecules, which damage resident cells needed for repair and tissue regeneration [77]. It has been described that, in response to an injury, stromal stem cells modify their phenotype, and some interleukins, such as IL-lβ, TNFα, and IFN-ɣ, have been shown to inhibit colony formation in vitro [78]. Similarly, bovine endometrial MSC have been shown to respond to lipopolysaccharide exposure through secretion of proteins mainly related to tissue remodeling, immune responses, and angiogenesis [79]. The extracellular matrix was described to interact with the local and/or systemic stem cell niche through growth factors, chemokines and other regulatory molecules that determine the quiescence state or proliferation, self-renewal or differentiation, migration or retention and death or cell survival [80]. During inflammation cells sense tissue damage by endogenous factors released to the extracellular milieu by dying or necrotic cells or extracellular matrix components and respond by producing specific molecules for recruiting neutrophils and mononuclear peripheric blood cells aimed to remove cell wastes [81]. Hence, the effect of inflammation upon tissue and cell responses is strictly related to the way it begins, maintains, and resolves the inflammation [82] (Figure 2).

Prostaglandin E_2_ (PGE_2_) has been demonstrated to play a critical role in guiding and governing various aspects of the inflammatory response, causing potent immunosuppressive effects that contribute to the resolution phase of acute inflammation and facilitating tissue regeneration and return to homeostasis [83]. It has been proposed that during inflammation, PGE_2_ generates cellular changes in the endometrial-tissue-mediated activation of resident stromal progenitor/stem cells [50]. There is evidence that, in vivo, PGE_2_ produced locally in response to tissue damage is necessary to enhance the proliferative effects of stem cells to promote organ repair [84]. 

A new role for PGE_2_ in hematopoietic stem cells (HSC) homing and survival, as well as short-term-HSC engraftment have been supported by pre- and clinical trials of blood transplants in human and primates [85,86]. Others have extensively studied the use of PGE_2_ in MSC research and applications [84,87]. Lee et al. [88], showed that PGE_2_ produced by MSCs contributes to maintenance of self-renewal capacity through EP2 receptor in an autocrine manner, and PGE_2_ secretion is down-regulated by cell-to-cell contact, attenuating its immunomodulatory potency. However, PGE_2_ is an unstable pH-dependent molecule with a relatively rapid degradation and short half-life [89]. Recently, 16,16-dimethyl prostaglandin E_2_ or (dmPGE_2_), a stable derivative of prostaglandin E_2_, originally identified in zebrafish [90] had been used as a new tool for the expansion of HSC in zebrafish and in mice and it is currently in preclinical and clinical trials in humans [91]. The use of dmPGE_2_ showed long lasting effects on the function of HSC without side effects such as over-proliferation. In other models, dmPGE_2_ stimulated the amplification of progenitor/multipotent cells during differentiation of murine embryonic stem cells. It was demonstrated in vivo [84], that the interaction of the PGE_2_/Wnt pathway was mediated by EP2 and EP4 receptors. PGE_2_ potentiated the effect of Wnt signaling during embryogenesis by stabilizing β-catenin, and it was required for the regulation of Wnt-mediated HSC development. In 2009, Hoggatt et al. [87], reported that ex vivo treatment of murine bone marrow with dmPGE_2_ improved the graft, probably through an improvement in HSC homing and survival. The improvement in HSC function was maintained, without additional treatment with dmPGE_2_, suggesting that there was a long-term effect on HSCs. These findings provide strong evidence that using dmPHGE_2_ could be an effective means of increasing the number of stem cells within a context of therapeutic use. Currently, use of dmPGE_2_ is consider among the novel strategies for improving hematopoietic reconstruction after allogeneic hematopoietic stem cell transplantation or intensive chemotherapy. However, to date no report on the use of dmPGE_2_ in endometrial stem cells had been published in any species. It will be interesting to assess the proliferation, migration, and immunomodulation of endometrial MSC pulsed with dmPGE_2_. 

We challenged cattle endometrial MSC in vitro with different concentrations of PGE_2_ and assessed the biological properties of primed cells and control, non-exposed to PGE_2_ cells [50]. The exposure in vitro of MSCs to PGE_2_ modifies their transcriptomic profile, covering mainly biological processes such as cellular component organization or biogenesis and cellular and metabolic processes, as well as biological regulation, development, growth, and the immune system. Thus, PGE_2_ may have a potential role in the fate of stem cell activation, migration, and homing processes during pathological uterine inflammation, such as in endometritis, and in the healthy puerperal endometrium [50].

#### 4.2.4. Mesenchymal Stem Cells and Endometrial Regeneration

Endometrial MSC are responsible for regenerating the endometrium in humans in a paracrine way regulated by estrogen [92] and involving the Hippo signaling pathway [93]. Manipulating human endometrial MSC is consider one alternative for treating endometriosis via inhibition of TGFβ1 signaling [94]. In mares, endometriosis is an incurable disease leading to huge economic loses for the equine industry. In this sense, cell therapies using MSC are foreseen as an attractive perspective based on the results obtained in the treatment of other inflammatory conditions. Research on eMSC and their potential use is new and the driving force for research on eMSC relied partially in the assumption that such cells will keep an epigenetic memory of the original tissue from which they were isolated, and thus will perform better if used in the treatment of uterine pathologies [50]. So far this has neither been proof nor tested, and all the published results using MSC to treat endometrial diseases used cells obtained from other niches (see below).

In mares, [71] infused adipose derived allogeneic MSC labeled with a fluorescent-nanocristal green dye (Vybrant CFDA) in the uterus of mares diagnosed with endometriosis, a chronic degenerative disease of the uterus, and found homing of the infused cells at day 7. Though no attempt were made to quantify the amount of homing, proliferation of both glandular and stromal cells was detected using Ki–67 quantifications. Interestingly, at day 60, authors showed positive remodeling of endometrial tissue of the mares with endometriosis. Whether this therapeutic action was mediated by MSC homing and proliferation, their paracrine effects or activation of resident (or migrating) populations of MSC was not addressed. 

We conducted a limited filed trial in 2015 in which six barren mares of scored infertility, with grade IIb endometriosis, and at least two years of unsuccessful mating, were infused with 20 million autologous eMSC per uterine horn. Three of the mares (50%) became pregnant and foaled (unpublished). However, larger double-blinded clinical trial should be performed to withdraw solid conclusions about the potential use of eMSC for treatment of endometriosis. Others have recently administrated MSC for different pathologies that course with fibrotic processes in the uterus of female mammals. This includes infusions of MSCs derived from bone marrow in human patients with Asherman´s syndrome [95,96] or through the recruitment of MSCs derived from bone marrow through CXCL12 in the same syndrome [97]. MSCs derived from bone marrow have also been used in rat models promoting the thickening of the endometrium [98,99], in intrauterine adhesions in rats [100,101], in endometrial fibrosis model in rats [102] and infusions of MSCs derived from umbilical cord in chronic endometrial damage in rats [103]. The use of such therapies in domestic species had been poorly explored. In mares MSCs derived from bone marrow were infused prior to insemination in healthy animals; however, no improvement of pregnancy rates was observed [104]. Using adipose MSC in mares with chronic endometritis [105], or in mares with endometriosis [71] had been reported. Very recently MSCs derived from endometrial tissue were infused in mares in diestrus [106]. It is worth noting that in the last example, no pathology was present in the endometrium and the only goal was to detect homing of labeled cells in the host endometrium. From the discussed above, it is starting to emerge that MSCs can be used as therapeutic options in equine reproductive medicine.

## 5. Conclusions and Future Directions

There is a growing interest in the study of the presence and functionality of endometrial stem cells in farm animals, due to their tremendous potential impact in reproduction and for the treatment of some reproductive diseases such as endometriosis in mares. Of relevant importance is also cystic endometrial hyperplasia in dogs and equine endometritis, as well as in hypoplasia or endometrial atrophy observed in several species [107]. Nevertheless, endometrial MSC might be of value in other fields of veterinary medicine, tissue engineering and regenerative medicine as it has been shown for human menstrual and endometrial MSC [17,108]. There is the need to fill the gap existing with similar research in humans and mice, and there are more questions than answers at present. The endometrium has been considered as an attractive, accessible, and renewable cell source for regenerative therapies. Further research is necessary in particular areas such as in vivo location of the niches, their immunomodulatory and anti-infective properties. Also, validation of the actual “stemness” of eMSC is mandatory.

It is generally accepted that the ultimate proof of stemness for any given stem cell type should be the repopulation of a tissue (or organ) previously depleted of cells. The gold standard for testing stemness of endometrial MSC would be reconstitution in vivo by xenografts of putative stem cells populations [39] in immunodeficient (NOG) mice. Cells are often transplanted into ectopic sites such as the kidney capsule or subcutaneous tissue, and although these sites do not resemble the microenvironment of the cellular niche tissue; they provide a rich vascular supply and contain the transplanted cells in a confined region [39]. Formation of well-organized endometrial and myometrial layers, comprising glands that proliferated in response to estrogen, stroma that differentiated into deciduous cells under the effect of exogenous P4 and formation of large bloody cysts after the withdrawal of hormones had been reported for human endometrial MSC [8,35]. No such models are available for other species. Therefore, testing actual stemness of farm animals endometrial stem cells is based on other biological attributes of MSC such as tri-lineage differentiation, migration, expression of surface markers and of profile of cytokine secretion. Therefore, ultimate demonstration of stemness for endometrial MSC of farm animals is still missing.

Finally, farm animal research on eMSC can be also of great value in translational research for certain uterine pathologies and for immunomodulation of local responses to pathogens, hormones, and other substances. Recently sheep have been proposed as an autologous model for preclinical research of in urological and gynecological diseases, such as pelvic organ prolapse and stress urinary incontinence [17]. 

## Figures and Tables

**Table 1:** Summary of studies that have identified possible candidates for endometrial stem cells in farm animals.

Species	Endometrial Stem Cell Candidates		Reference
	Type	Characteristics	
Swine	MSCs	MSC marker expression Osteogenic and adipogenic differentiation	[11]
Swine	SP	Embryonic and mesenchymal marker expression Chondrogenic and osteogenic differentiation	[43]
Sheep	MSCs	CD271^+^ CD49f^−^ population with high clonogenic efficiency, serial clonogenic ability and differentiation into adipogenic, myogenic, chondrogenic and osteogenic lineages	[13]
Goat	MSCs	High proliferation potential and differentiation into adipogenic, chondrogenic and osteogenic lineages	[14]
Bovine	Stromal	Bone marrow mesenchymal cell-like phenotype Ability to differentiate into osteogenic lineage	[44]
Bovine	Progenitor/mesenchymal	Pluripotency and multipotency marker expression Multilineage differentiation, clonogenicity and high proliferation abilities	[12]
Bovine	MSCs	CD73+ marker expression Differentiation into adipogenic lineage and high proliferation ability	[45]
Bovine	Pluripotent	Pluripotency marker expression Multilineage differentiation ability	[46]
Bovine	MSCs	Mesenchymal marker expression Clonogenicity Differentiation into osteogenic and adipogenic lineages	[47]
Bovine	MSCs	Fibroblast-like morphology and adherence to plastic Multilineage differentiation, alkaline phosphatase activity, clonogenicity and high proliferation ability Pluripotency and multipotency marker expression	[48]
Equine	MSCs	Fibroblast-like morphology and adherence to plastic Multilineage differentiation, fast doubling, and migration abilities Expression of CD44, CD90 and MHCI surface markers	[15]

MSCs: Mesenchymal stem cells; SP: Side population.
